# Bioactive Nanocomposites for Tissue Repair and Regeneration: A Review

**DOI:** 10.3390/ijerph14010066

**Published:** 2017-01-11

**Authors:** Jane Bramhill, Sukunya Ross, Gareth Ross

**Affiliations:** Biopolymer Group, Biomaterials Center of Excellence, Department of Chemistry, Faculty of Science, Naresuan University, Phitsanulok 65000, Thailand; janebramhill@me.com (J.B.); sukunyaj@nu.ac.th (S.R.)

**Keywords:** nanocomposites, bioactive materials, bone regeneration, tissue repair

## Abstract

This review presents scientific findings concerning the use of bioactive nanocomposites in the field of tissue repair and regeneration. Bioactivity is the ability of a material to incite a specific biological reaction, usually at the boundary of the material. Nanocomposites have been shown to be ideal bioactive materials due the many biological interfaces and structures operating at the nanoscale. This has resulted in many researchers investigating nanocomposites for use in bioapplications. Nanocomposites encompass a number of different structures, incorporating organic-inorganic, inorganic-inorganic and bioinorganic nanomaterials and based upon ceramic, metallic or polymeric materials. This enables a wide range of properties to be incorporated into nanocomposite materials, such as magnetic properties, MR imaging contrast or drug delivery, and even a combination of these properties. Much of the classical research was focused on bone regeneration, however, recent advances have enabled further use in soft tissue body sites too. Despite recent technological advances, more research is needed to further understand the long-term biocompatibility impact of the use of nanoparticles within the human body.

## 1. Introduction

The unique properties of nanocomposites have attracted notable research interest, especially in bioapplications owing to their structural features. The different types of nanocomposites—organic–inorganic, inorganic–inorganic and bioinorganic nanomaterials—have allowed their use in biomedical fields such as drug delivery, cancer therapy, medical imaging, and chemical sensing. A nanocomposite is defined as a multiphase solid material in which one of the phases has one, two, or three dimensions less than 100 nm [1]. These materials may be both artificially generated or of natural origin, such as the shell of a snail or mollusc. This is an example of an organic–inorganic nanocomposite, where the inorganic phase of the shell is based on calcium carbonate and the organic phase on the proteins perlucin and conchiolin. The aim of this review paper is to focus on the main applications of bioactive nanocomposites, with particular focus on tissue repair and regeneration. First it is important to establish the terminology of bioactivity and discuss the classification of nanocomposites. Then the review will discuss the applications related to that specific area of research.

## 2. General Nanocomposite Information

**Figure 1:**
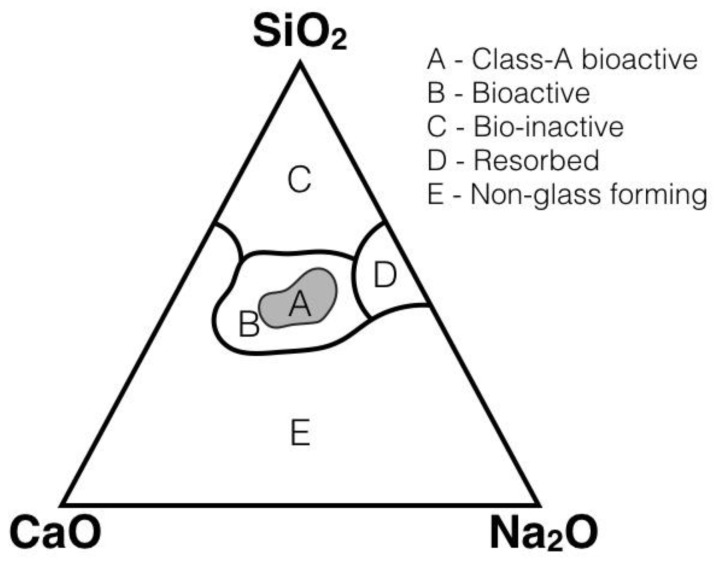
Property changes of bioglass materials (adapted from Tilocca [5]).

Nanocomposite bioactive materials are suitable for both bone and tissue applications, due to the many biological interfaces and structures functioning at the nanoscale. The terms tissue repair and regeneration can be used interchangeably, in particular when in reference to wound healing. However, in the correct usage, in repair deposition of connective tissue forms a scar allowing the injured tissue to return to normal functioning whilst in regeneration uninjured tissues within epithelia are able to repair tissue to function as normal without scar formation, both mechanisms resulting in a similar outcome [2]. The terminology for bioactive materials can be dated to 1969, when Hench defined the concept a bioactive material as “one that elicits a specific biological response at the interface of the material which results in the formation of a bond between the tissues and the material” [3] (Figure 1). Hench provided early criteria for the specification of bioactive materials, which was used up until 1994 when a new two-classification system was proposed [4]. Class A materials are osteoproductive, they possess a bioactive surface, which can be colonized by osteogenic stem cells due to the material inducing both an intracellular and extracellular responses. An example would be 45S5 Bioglass. Class B are osteoconductive, providing a biocompatible surface for bone migration. This material type, an example of which is synthetic hydroxyapatite (HA), induces only extracellular responses from the target tissue.

What is apparent from the above classification is that bioactive materials have strong links with bone regeneration. Bioactivity can also relate to tissue engineering scaffolds with the goal to create scaffolds that combine both bioresorbable and bioactive properties that can activate in vivo mechanisms of tissue regeneration, inducing stimulation for the body to heal itself and which then leads to replacement of the scaffold by the regenerated tissue [6]. The interaction encountered with a nanocomposite can be far more efficient than possible with macro/micro structured alternatives, with bioactive nanocomposites typically being used in one of two ways, either as a thin biocatalytic coating or as a bulk product. For example, 10 wt % bioactive glass nanoparticles can provide a significantly greater number of sites for hydroxyapatite nucleation compared to 10 wt % micron-sized bioactive glass particles [7]. Loher et al. [8] demonstrated that the enhanced specific surface area of nanoparticles improve the degradation and bioactivity compared to micron-sized particles. In the context of bone tissue engineering, nanocomposites more closely mimic the structure of natural bone, which can be represented as a highly hierarchical nanocomposite involving nanoscale HA crystallites and collagen.

In nanocomposite research there are a number of choices for the components that comprise the composite. Classically they are characterized into the three following categories depending on the matrix or continuous phase: ceramic-, metallic-, and polymer-based nanocomposites, although with the advances in graphene technology in producing nanocomposites a 4th category may be required. However, this review paper will continue to use the classic classification system and highlight when carbon matrix composites are used. Ceramic-based nanocomposites have advantages over their conventional counterparts in the form of increased strength, hardness, and abrasion resistance, achieved by refining particle size, along with enhanced ductility, formability and superplasticity due to the nanophase. They can also exhibit a change in electrical conduction and magnetic properties by increasing the disordered gain boundary interface. Metallic-based nanocomposites also have increased hardness, strength and superplasticity; they also tend to have a lowered melting point. Again the electrical and magnetic properties are altered and metallic nanocomposites can display improved magnetic properties such as coercivity, superparamagnetism, saturation magnetization and magnetocolatic properties. The last group considered in this review are polymer-based nanocomposites, which can possess various properties such as catalytic, magnetic and or optical functions, which are normally due to the inorganic nanocomponent. The polymer matrix component provides the needed mechanical and thermal stability to the structure of the nanocomposite. Biocompatible polymers such as polyvinyl alcohol (PVA), polyethylene glycol (PEG), polycaprolactone (PCL), and polyvinylpyrrolidone (PVP) have attracted much attention for use in biomedical applications due to their mechanical properties, thermal stability, permeability, optical transparency and degradation rates. There has also been significant research using biodegradable polymers, mainly focused on poly(lactic acid) (PLA) and its copolymers.

The preparation of nanocomposites will be discussed within each classification. Ceramic-based nanocomposites can be prepared using various methods, but the sol-gel technique is one of the most commonly used methods to prepare inorganic bioactive nanoparticles. The bioactive nanocomposites can be embedded in a sol-gel matrix, and the embedded material retains its conformation and chemical/physical properties, whilst the matrix allows external reagents to be transported to the embedded biomolecules to allow chemical reactions and interactions to occur. Sol-gel processing is important in controlling the shape and morphology of bioactive glass nanoparticles by varying the synthesis parameters [9,10], with microemulsion and gas phase being amongst the most commonly used techniques for their synthesis. For the production of inorganic nanofibres, laser spinning can be used, in particular to produce bioactive glass nanofibres [11].

Metallic matrix nanocomposites can be prepared by a number of techniques, but some of the most common are the following: spray pyrolysis, rapid solidification, vapour techniques; (physical and chemical vapour deposition), electrodeposition and chemical methods, which include similar processes to ceramic (colloidal and sol-gel) manufacture and ball milling [12,13]. Although important for many applications, for example metal matrix nanocomposites may be used as hyperthermia agents that via heating deliver thermal energy to a target tissue such as a tumours [14], their use in tissue repair and regeneration has limitations and specific properties that do not lend themselves for this particular application. Therefore, discussion of metal matrix nanocomposites in this paper will be limited.

**Figure 2:**
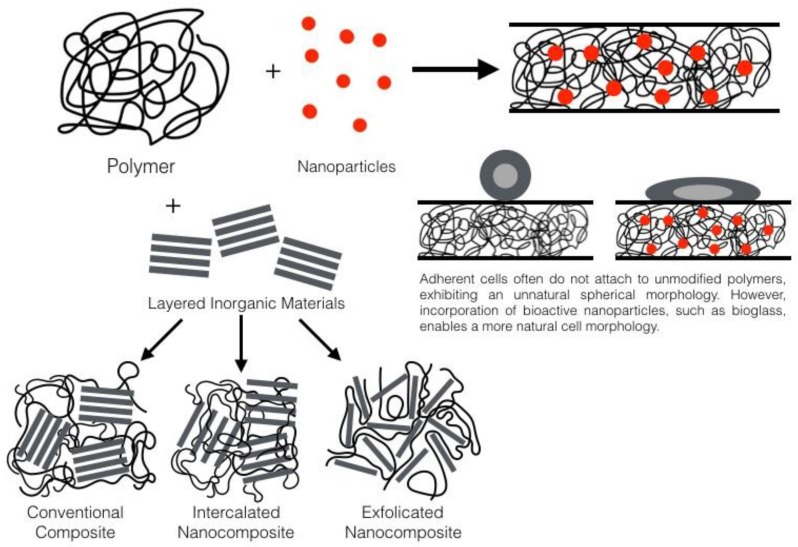
Two types of polymer-based nanocomposites.

Polymer matrix nanocomposite materials can be prepared via the addition of an inorganic material to a polymer matrix, for instance adding nanoparticles, fibres, or clays (Figure 2). Nanoparticle size fillers have a large surface area and due to their size they are able to form an intimate interface with the polymer matrix within the composite. This gives this type of material advantageous properties and higher performance (e.g., mechanical properties) than expected [15].

In the case of polymer composites containing bioactive glass filler particles, a direct comparison between micron-scale bioactive glass and nanoscale bioactive glass composites has been carried out using poly-3-hydroxybutyrate (P(3HB)) as the biopolymer matrix [16]. It was shown that addition of bioactive glass nanoparticles significantly improved the mechanical properties, hydrophilicity, and protein adsorption, while producing a nanotopography suitable for enhanced cell growth. The P(3HB)/bioactive glass nanocomposites also showed cytocompatibility towards MG-63 osteoblast-like cells. Another example was shown by Pramanik et al. [17] who added HA nanoparticles to poly(ethylene-co-acrylic acid) and observed a significant increase in modulus and strength of the material in comparison to HA microparticles. In terms of the microstructure of nanocomposites, Wei and Ma [18] showed that nano-HA particle reinforced poly(L-lactic acid) (PLLA) composite scaffold exhibited a better dispersion of HA, which led to superior mechanical properties of the scaffolds compared to those with similar concentrations of micron-sized HA particles.

The advantages of nanocomposites in cell adhesion (Figure 2) were also reported by Webster et al. [19] who stated that osteoblast, fibroblast, and endothelial cell adhesion on nanophase materials (titania, alumina, hydroxyapatite) was significantly greater than that seen on conventional formulations of the same ceramic materials. Notable was an increased adsorption of vitronectin on the nanophase material, which may facilitate enhancement of the adhesion of osteoblast cells. In addition the analysis of a nanophase calcium phosphate-doped poly(lactic-co-glycolic acid) scaffold suggested much improved bioactivity, degradation rate and mechanical properties compared to that of the pure polymer [19].

Although the use of nanoparticles in biocomposites has numerous advantages, the agglomeration of nanoparticles during processing remains an important issue to address [20]. A poor dispersion of nanoparticles within the polymer solution or melt leading to agglomeration will negatively affect the final distribution of the nanoparticles in the matrix leading to materials with low mechanical properties. In addition, a cluster of nanoparticles may prevent a proper interface with the polymeric matrix thus increasing the brittleness of the nanocomposite [21].

## 3. Bone Tissue Engineering

The advantages seen with bioactive nanocomposites in the previous section demonstrate the potential of these types of materials. One area where there is great potential for their use is in the treatment of critical size bone defects that occur due to trauma or disease. This treatment would be based on tissue engineering approaches involving the use of engineered porous structures known as scaffolds. Scaffolds for bone tissue engineering are made of suitable (bioactive and biodegradable) bioceramics, polymers or combinations of them thus forming composites [22]. The interactions of the following four proteins (fibronectin, vitronectin, laminin and collagen) in bone regeneration are known to enhance osteoblast function. Nanomaterials have displayed greater integration of all these proteins as opposed to regular sized materials [19], therefore, with this and the advantages of cell growth and adhesion reported with nanocomposites, research into their use in bone regeneration was investigated.

The mode of action of inorganic bioactive materials designed for bone tissue engineering is based on their high surface reactivity. When they come into contact with physiological fluids strong bonds are formed with the bone via biological interactions [23]. An alternative method involves the formation of electrostatic chemical bonds that can occur between a positively charged amine group and a negatively charged oxygen surface of a ceramic. Lysine, hydroxylysine, histidine, and arginine are all positively charged amino acids, which can participate in this type of reaction. Secondary types of chemical bonding could also occur, such as hydrogen bonding from hydroxyl to carbonyl groups, or negatively charged surfaces have the potential to attract organic components. [3]. Therefore, in summary the regeneration of bone requires the follow criteria [24]:

- Osteoinduction
- The presence of cells to respond to osteogenesis signals
- A scaffold material to support the growth of cells, osteogenesis and subsequent ECM formation
- Vascularisation of the treatment area

The need to have a host response and the early findings led to further research in this area focusing on bioactive glass and glass ceramics due to their ability to chemically bond with living bone tissue. In order for this bond formation to occur, a layer of biologically active hydroxycarbonate apatite (HCA) forms on the surface in response to exposure to physiological fluids [3,25]. The HCA crystals become reinforced by collagen fibres, which form bonds between the bioactive material and the tissue [3]. As the structure of natural bone consists of 70% inorganic component-apatite and 30% organic component collagen [26], it is logical to see why bioglasses have become the accepted treatment for bone repair.

**Figure 3:**
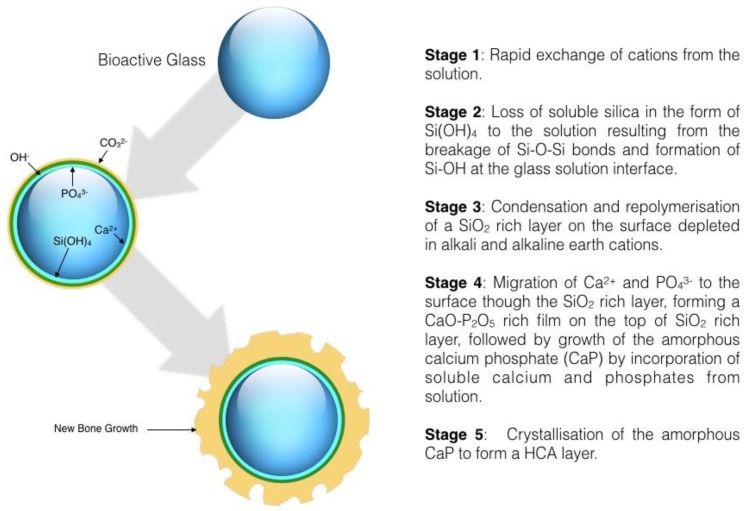
Bioactive glass surface reactions.

The first bioactive glass developed by Hench et al. in 1969 [3] was Na_2_O-CaO-P_2_O_5_-SiO_2_. Hench described in detail the reactions that occur during the bond formation, which in relation to SiO_2_-CaO-P_2_O_5_ Sol-Gel glasses is represented by the five steps shown in Figure 3 [27,28].

There are now many more bioactive glasses/glass ceramics [29,30] as well as sintered hydroxyapatite (Ca_10_(PO_4_)_6_(OH)_2_) [31]. Bioactive glasses are silicate-based amorphous materials which properties change with composition, as shown in Figure 1. Bioactive glasses become coated with a layer of apatite as a response to contact with physiological fluids, and this layer then acts to promote the formation of new bone. In vitro studies have shown that apatite formation depends on several factors [32,33,34], including surface area and porosity [35,36].

When silica-based bioactive glasses and ceramics are used, a partial dissolution of the network at the surface occurs, followed by a polycondensation reaction which induces the formation of a layer of silica on the surface, following this process a layer of calcium phosphate is formed [37]. Focusing on CaO-P_2_O_5_-SiO_2_ gel glasses it has been demonstrated in vitro that the formation of apatite increased when the CaO content was increased [32], CaO and P_2_O_5_ increased in non-devitrified glasses [38], and when CaO increased and P_2_O_5_ decreased this resulted in a high pore volume and surface area effects [38]. High CaO content in gel glasses increases their pore volume, therefore decreasing the surface area [39,40,41]. This discovery led to the development of a family of CaO-P_2_O_5_-SiO_2_, CaO-P_2_O_5_-SiO_2_-MgO bioactive glasses produced by sol gel processing [32,42,43,44].

Hydroxyapatite (HA), the synthetic version of bone apatite, is one of the most important biomaterials in the bone tissue engineering field. Recently, the combination of HA with chitosan (CTS, which has excellent biocompatibility), has made these two materials most important for this application [45]. Chitosan is a natural based-polysaccharide, the *N*-deactylation product of chitin, in which glucosamine and *N*-acetylglucosamine units, are connected via β-(1,4) linkages. It is biodegradable, does not induce an immune response during use or degradation and has advantageous mechanical properties, making it a widely used biomaterial for several applications [46]. CTS on its own displays low osteoconductivity, so in order for it to be suitable for bone tissue engineering applications, the addition of HA is needed to provide sites for calcification, thus increasing the osteoconductive behaviour of the material. Depan [47] produced a chitosan-based hybrid network nanocomposite. The chitosan was grafted onto propylene oxide forming a modified chitosan which was then bonded to ethylene glycol-functionalised nHA, resulting in a highly porous structure. The scaffold supported adhesion, proliferation and viability of osteoblast cells, which infiltrated and re-colonised the pores of the scaffold.

Peng et al. [48] fabricated HA/CTS nanofibrous scaffolds to assess whether they supported the osteogenic differentiation of murine mesenchymal stem cells (mMSCs) compared to pure CTS scaffolds. The mMSCs were cultivated on the nanofibrous meshes in the presence and absence of osteogenic medium for 7 days and the changes in morphology, population growth, osteogenic gene, and protein expression of the seeded cells were observed. Imaging showed mMSCs adhered and spread significantly more on the HA/CTS scaffolds than on the pure CTS scaffolds. Crucially, the cells seeded onto the nanocomposite scaffolds that were cultured in the absence of osteogenic medium were found to have an increased transcription and expression of genes thought to be involved in osteoblast maturation (Collagen I, Runx2, ALP and OCN) as well as exhibiting bone marker proteins characteristic of osteoblastic differentiation (ALP and Collagen I) compared with cells seeded onto pure CTS scaffolds cultured under the same conditions. When the seeded nanofibrous scaffolds were placed in osteogenic medium, the osteogenic differentiation of mMSCs was potentiated by the osteogenic supplementation and thus these cells exhibited an earlier and higher expression of the aforementioned genes and bone marker proteins.

These results show that the HA/CTS nanocomposite scaffolds are able to support mMSCs differentiation into osteogenic lineages without osteogenic supplementation and outperformed pure CTS scaffolds in terms of cell growth, proliferation, attachment and differentiation. Thus, the nanocomposite scaffolds were proven to be osteoinductive and osteoconductive, making them very attractive options for bone tissue engineering, although further investigation including in vivo testing is needed.

In order to achieve a nanoscale bone-like structure, Kim et al. [49] exploited a processing route that involved the preparation of a HA-gelatin nanocomposite solution by biomimetic precipitation to ensure a uniform distribution of the HA nanoparticles within the polymer matrix. To assess the biocompatibility of the nanocomposite fibres, bone-derived cells (MG63) were seeded onto them and cultured for 7 days. The cells exhibited a normal morphology and intimate contact with the nanofibres. Measurement of alkaline phosphatase (ALP) activity expressed by the cells revealed a significantly higher bone-forming activity of cells seeded on the HA-gelatin nanocomposites compared to the pure gelatin nanofibres, thus demonstrating an improvement of the osteoblastic activity on the nanocomposite fibres.

The utilisation of biodegradable polymers in biodegradable nanocomposites has great potential as tissue repair and/or regeneration in orthopaedics. However, in order to provide and maintain suitable mechanical properties for specific target applications, the filler content of the composite is usually low. A higher content may result in reduced biomimetic mechanical behaviour. This poses a problem, as a low volume fraction of hydroxyapatite within biodegradable composites may not support sufficient successful bone growth. There are therefore several factors to consider when developing nanocomposites for bone regeneration [50]. Wei and Ma [18] investigated nano-hydroxyapatite scaffolds for bone tissue engineering with a view to a better mimicry of the mineral component and microstructure of natural bone. The nanohydroxyapatite (nHA) scaffolds were prepared using thermally-induced phase separation. The nHA particles were dispersed and bound into the pore walls of PLLA scaffolds, presence of nHA significantly improved both the protein adsorption and mechanical properties of the scaffold. Prabhakaran [51] also investigated a range of PLLA/nHA scaffolds. These were PLLA only, PLLA/nHA, and PLLA/collagen/nHA. Osteoblasts adhered and grew selectively on PLLA/collagen/nHA scaffold with an enhanced mineral deposition 57% higher than on the PLLA/nHA scaffold. This suggests a synergistic effect of the presence of collagen and nHA within the scaffold and the presence of an extracellular matrix (ECM) protein. This provided both cell recognition sites and apatite for cell proliferation and osteoconduction, which is necessary for mineralisation and bone formation.

Jayabalan [50] investigated a hydroxyl-terminated high molecular weight poly(propylene fumarate) thermoset with hydroxyapatite filler. Cross-linked poly(propylene fumarate) is able to provide mechanical support and scaffolding for cell attachment to the treatment area whilst it degrades slowly. It is bone osteogenic and osteoconductive enabling fast osteointegration at the defective bone area. Aggregates of calcinised HA nanoparticles with a rod shape within the polymer matrix facilitated interfacial bonds and improved mechanical interloading.

Bioglasses have also been used with biodegradable polymers, Hong et al. [10,52,53] synthesised 3D porous PLLA-bioglass nanocomposite scaffolds that contained varying concentrations of bioglass nanoparticles prepared via the sol-gel process. In vitro bioactivity was favoured in the scaffold containing 20% wt bioglass. The scaffolds also displayed an increase in the compressive modulus from 5.5–8 MPa with 0%–30% bioglass content by weight. Jo et al. [54] reported on the mechanical properties and biocompatibility of bioglass materials prepared via a sol gel method specifically reporting on the effect of aspect ratio of polycaprolactone (PCL)/bioglass. The nanocomposite and microcomposite sized PCL/bioglass scaffolds were compared. With 20% weight bioglass filler the nanocomposite material had significant improvement biocompatibility and elastic modulus with only a small reduction in tensile strength, when compared to the microcomposite material. In addition to the enhanced biocompatibility in vitro, the osteoblast activity was also improved. Nanocomposites formed from biodegradable PCL and superparamagnetic iron doped hydroxyl apatite (Fe/HA) were prepared by Banobre-Lopez [55]. They discovered that an inclusion of 10% magnetic oxide nanoparticles (MNPs) increased the strength of the PCL network, however when ratios of 70:30 and 80:20 PCL:FeHA were investigated, a reduction in strength and an increase in brittle behavior was observed. The PCL/FeHA materials were prepared using 3D bioplotting to form a layer by layer structured scaffold which displayed good cell adhesion [14,55].

Graphene and its compounds display unique chemical and physical properties and due to these unique advantageous properties graphene oxide (GO), reduced graphene oxide (RGO) and GO-nanocomposites have recently attracted interest in a wide range of areas in many fields, including biomedical applications, as they are superior nanoparticles for use in medical applications due to their superior mechanical and electrical properties [56,57]. Chen et al. [58] investigated a zinc oxide/carboxylated graphene oxide (ZnO/GO-COOH) nanocomposite with a view to assessing the effect of the addition of the carbonyl group and its ability to promote osteogenesis. The release of zinc ions and antibacterial activity against *S. mutans* was also assessed. It was discovered that CO-COOH has greater osteogenic activity in comparison to previous studies involving carbon nanotubes [59,60]. However high levels of graphene oxide concentration did induce cytotoxicity, which is similar to the findings with graphene [61]. Toxicity depends on a multitude of factors such as particle size, type and degree of functionalisation and the particular cell type under investigation. The attachment of ZnO nanoparticles to the GO-COOH sheets resulted in a sustained release of ZnO without a burst release; this is advantageous as it has the potential to prevent cytotoxic effects. Chen et al. [58] were able to demonstrate the up-regulation of ostegenic gene expression upon contact with the ZnO/GO-COOH nanocomposite, proving a potential synergistic osteogenic effect. Furthermore, ZnO/GO-COOH nanocomposites increased the surface water wettability and surface roughness compared to the control material. This is highly advantageous as hydrophilic surfaces enhance the binding of adhesive proteins and can also enhance the proliferation and differentiation of osteogenic cells. Another advantage of the zinc ions and GO system is that they are known to have antibacterial effects [61,62]. The sharp edges of the GO-COOH sheets [63] and the surface abrasiveness of ZnO [64] are capable of physically damaging the bacteria, thus providing sufficient antibacterial effects.

Ideal bone tissue engineering materials used in other body sites require different bioactive properties, for example, dental implants should be capable of initiating both osteoinductivity and osteoconductivity [65]. The material should have sufficient surface topography to enhance osteoconductivity, however, the potential for bacterial colonization of the surface should be understood [66]. The implant should also possess long-term durability, sufficient mechanical properties, and resistance to saliva corrosion [67]. If possible the support of bone ingrowth is an advantageous property, particularly in diabetic patients [68]. Titanium and its alloys are the most accepted commercial dental implants. They are biocompatible and have ideal mechanical properties [69].

Osteogenic growth peptide (OGP) is a naturally occurring growth factor peptide important to the ECM. The peptide is short, linear, and present in serum at μmol/L concentrations [70]. OGP is a soluble peptide that in vitro regulates the differentiation of bone marrow mesechymal stem cells (BMScs) into osteoblasts [71]. When administered intravenously repeatedly, OGP has been shown to also improve fracture healing [72]. Chen et al. [73] investigated the formation of a mineral/OGP nanocomposite layer on titanium and the effects this had on MSCs, using the presence of alkaline phosphotase (ALP) as a key osteogenic marker. MSCs were present on both the mineral control and the mineral/OGP surface. There was a significant increase in the number of MSCs seeded at day 5 on the mineral/OGP surface, but by day 7 there were no longer differences between control and OGP containing surface. This may be due to a transition of the osteogenic phase from proliferation phase towards the osteogenic differentiation phase. ALP levels released from MSCs, measured for the mineral/OGP layer, were significantly higher than that of control material. However, with an increase in test period, there was found to be a greater level of ALP present in the positive control material. This further supports the suggestion that combination of mineral/OGP facilitates the progression from osteogenic proliferation towards differentiation.

## 4. Nanocomposite Wound Dressings

Bioactive scaffolds have the ability to mimic natural extracellular matrix (ECM) acting as a template for cells to adhere, multiply, migrate and function. The scaffold provides points for cell attachment, and allows the delivery of both bioactive agents and growth factors, while the scaffold material should also supply mechanical stability to cells and surrounding tissue [74]. As so many potential benefits depend on the suitability of the scaffold, it is useful to have the mechanical properties of the target tissue in mind when designing and manufacturing a material [75,76]. The materials are usually based on hybrid inorganic/organic, bioactive/polymer nanocomposite materials. One of the most common needs for tissue engineering scaffolds is in the treatment of wounds. An ideal wound dressing or scaffold material would: (a) be biocompatible; (b) elastic, and therefore comfortable to apply to the wound; (c) maintain a moist environment or provide moisture to a desiccated environment, whilst absorbing fluid and exudate; (d) stimulate wound healing and re-epithelialisation to reduce wound surface necrosis; (e) and prevent or protect from infection [77].

In addition to the above criteria for wound healing the development of new materials should be able to control or prevent microbial colonization which are important features are important due to emerging infectious diseases, which have significant implications for the economy and public health. There has been a significant increase in the number of infections developing in wounds that have become resistant to treatment from a multitude of drugs. This has increased the need for a new generation of wound dressings that are capable of addressing this problem. A potential solution under investigation may be the development of a combination of hydrogel and electrospun fibre scaffolds structured to mimic the natural ECM [78]. Avoiding further wound infection and angiogenesis stimulation are currently the greatest challenges in wound care management.

Composites based on the use of hydrocolloids have been investigated for potential applications as bioactive materials in the medical field [79,80]. Bioactive materials in wound dressings have active influences on the infection prevention and inflammatory, and subsequent healing processes [81]. The methods of producing such scaffolds from bacterial cellulose and hydrocolloids such as guar gum and or hyaluronic acid, include freeze casting, hydrocolloid mineralisation, biomineralisation by diffusion, hydrocolloid freeze drying, nanofibre electrospinning and direct injection [82].

Bacterial cellulose has been used widely in the production of bioactive wound dressings [83,84,85]. Bacterial cellulose has a high fluid permeability due to its highly porous structure which is advantageous for cell adhesion and subsequent proliferation. Its high water uptake capability can result in water contents greater than 90% which then provides a moist environment to the wound and absorbs exudate by interacting on a nanoscale with the wound surface [86]. These dressings accelerate and facilitate the healing process, reduce pain, and reduce the amount of care and monitoring of the wound needed. In burn victims the use of bacterial cellulose membranes facilitates the clearance of necrotic tissue and accelerates the re-epithelisation process [83]. Woehl [81] investigated bacterial cellulose/guar gum/hyaluronic acid biocomposites. A suspension of bacterial cellulose was mixed with a dispersion of 1:1 guar gum: hyaluronic acid, then, after film formation, collagen was deposited on the surface via a dipping procedure. The bionanocomposites are able to retain a high water content despite being a non-crosslinked hydrocolloid, and they are also stable in physiological conditions. Liquid uptake was shown to be dependent on the hydrocolloid content within the bionanocomposite, this allows for adjustment of the swelling behaviour of these materials. The low toxicity of these materials suggests they are suitable for future use with physiological media.

There are many advantages for using a hydrogel-based scaffold as a treatment for chronic wounds, and in particular an advantage is their ability to keep the wound bed moist. However due to this moist environment microbial infections in the wound cannot be prevented. *S. aureus* and *P. aeruginosa* are two of the most prevalent bacteria cultured from chronic wound environments such as leg ulcers [87]. Metallic nanoparticles have been widely studied as antibacterial agents due to their recognized toxicity against bacteria, yeast and some virus. These biological properties depend on the metal, size, structure, and large surface of the nanometric particles. Metal oxide nanoparticles such as ZnO, TiO_2_, CeO_2_, MgO and CaO have been investigated as inorganic antibacterial agents, although the majority of research is currently centered on copper and silver.

Silver has been used as an antibacterial/antimicrobial agent for many years. In order to provide antimicrobial action, silver must be accessible in a soluble form, which means either Ag^+^ or Ag°. Ag^+^ is typically used in a compound form, for example silver nitrate, whereas Ag° denotes crystalline metallic silver [88]. Due to their soluble nature, the silver particles in solution are taken up into bacterial cells where they subsequently interfere with DNA formation and replication, thus providing a bactericidal effect. Based on their historical and varied use silver-nanoparticles (AgNPs) have proven low systemic toxicity, they have also been shown to have synergistic effects in addition to their antibacterial properties such as anti-inflammatory behaviour.

In addition to the antimicrobial activity provided by AgNps there may be additional benefits to the wound healing cascade. The sequence of events that initiate re-epithelialisation and subsequently control the process is mediated by matrix metalloproteinases (MMP). Upon treatment of wounds with nanocrystalline silver, levels of inflammation were reduced; this was reported to be due to a cascade effect of a decrease in MMP. This subsequently lowered the number of inflammatory cytokines present in the wounds, induced apoptosis of neutrophils and thus decreased the level of TGF-β [89]. Liu et al. [90] used a surgical model to investigate the effect of silver on wound healing without the complications of burns or infection. Wounds treated with silver closed significantly quicker in comparison to control wounds. This reduced time to wound closure suggests that the presence of silver enhances the re-epithelialisation process by inducing the migration and proliferation of keratinocytes. This was observed alongside a reduction in formation of hypertrophic and keloid scar tissue suggesting that the AGNPs reduce the formation of fibroblasts or enhance the differentiation of the fibroblasts into myofibroblasts [90].

Bhowmick and Koul [91] investigated a hydrogel scaffold material that included AgNPs as an antimicrobial active material. The hydrogel was based on poly(vinyl alcohol) (PVA), a suitable material capable of providing the required moist environment to the wound area. The scaffold formed a reservoir which could be saturated with AgNPs to release into the wound bed. β-d-Glucose and starch were used to synthesise the AgNPs, and the presence of hydroxyl groups within starch is believed to facilitate molecular complexation of the silver ions within the nanoparticle structure which may improve the kinetics of particle release into the wound environment [91]. The scaffold was able to maintain a moist wound environment for 96 h, whilst exhibiting a sustained release of AgNPs.

Other studies on silver showed that incorporation of 20 nm silver nanoparticles in gelatin/HA nanocomposite films can exhibit strong bactericidal properties against various Gram-negative and positive bacteria [92]. While, Usman et al. [93] investigated polymer nanocomposite films, (PVA/GO/starch/Ag films) which were successfully prepared by one pot synthesis in a biological autoclave where PVA acts as matrix and starch as a green reducing agent. SEM results showed complete and uniform mixing of GO in PVA matrix while AgNPs were also synthesized and found to be well dispersed along with GO sheets in PVA matrix. Considerable improvements were observed in the mechanical properties and thermal behaviour of the polymer nanocomposites films. In case of PVA/GO/Ag/starch, the tensile strength and modulus were increased by 26.81% and 145%, respectively whereas thermal degradation temperature was increased ∼29 °C as compared to neat PVA. Moreover, these nanocomposite films also demonstrated enhanced antimicrobial potential due to synergistic effect of GO and AgNPs.

There are currently already products that use silver on the nanoscale for antimicrobial purposes. Acticoat™ and its variations are commercially available silver-containing wound dressings. The dressings are based upon a polyester core in-between layers of polyethylene coated with AgNPs. Silver is released from the dressing upon exposure to water, requiring the dressing to be soaked before application to the wound. This release of AgNPs initially occurs rapidly but then slows to a steady state release [88].

A new development in the generation of an antibacterial wound dressing involves the use of a copper containing bioactive glass nanocoatings. This follows the increase in the incorporation of inorganic ions into biomaterials due to their ability to stimulate angiogenesis and differentiation of stem cells for bone regeneration [94,95]. Studies have demonstrated that the antibacterial properties of copper nanoparticles associated with the release of Cu^2+^, are directly related to size. It has been observed that ion release from nanoparticles (diameters around 10 nm) embedded into polypropylene matrix increases quickly exhibits a high maximum release of particles during the first day; meanwhile, in microcomposites (diameters around 45 μm), the release rate gradually increases, releasing ions over a longer time period. The antibacterial behaviour of nanocomposites containing 5 *v*/*v* % of copper is able to reduce the concentration of *S. aureus* in 99.8% after 60 min, while microcomposites showed lower activity at the same time [96].

Other recent research on nanocomposite materials in this area have shown that some properties of wound dressings comprising polymers and gels can significantly improve by the addition of organoclay. Kokabi [97] investigated polymer-clay nanocomposite PVA hydrogel wound dressings with reinforcing agents such as Na-montmorillonite. It was observed that the quantity of clay added to the hydrogel nanocomposite had a direct effect on the properties of the wound dressing, specifically its effectiveness in the wound environment.

It has also been reported that HA nanoparticles possess an extraordinary adsorption of proteins as previously mentioned, due to the surface charge and particle texture [98]. Protein-binding surfaces of biomaterials are favourable for the platelet adhesion and the blood clotting formation due to the adsorbed proteins in blood coagulation [99]. HA exhibits both acid (PO_4_^3−^ ion) and base (Ca^2+^ ion) active sites based on its crystal structure [65]. The Ca^2+^ ion is an important factor in blood coagulation cascade, termed the fourth clotting factor, and is the only inorganic clotting factor [100]. It has been reported that the addition of Ca^2+^ ions not only decreases the induction period of the fibrinogen-fibrin system in the coagulation cascade, but also greatly promotes the fibrin monomer polymerization [101]. Microspheres fabricated from synthetic biodegradable polymers such as PLLA, and copolymers with polyglycolide (PLGA), and poly(ε-caprolactone) (PCL) are widely studied for tissue engineering and drug delivery. Song et al. [102], in order to develop new hemostatic agents with satisfactory properties, surface-modified the propertied of porous microspheres by fabricating hydroxyapatite nanoparticle graft-poly(d,l-lactide) (nHA-g-PDLLA) nanocomposites. The hemostatic properties of these porous microspheres were evaluated by platelet adhesion and whole blood clotting tests. The organic PDLLA copolymer component mainly functions as a skeleton to provide a large surface area for coagulation, while the inorganic HA nanoparticle component provides hemostatic activity. The alkaline treatment of nHA-g-PDLLA microspheres leads to the hydrolysis of the ester bonds of PDLLA chains, while the HA nanoparticles remain stable. Alkaline treatment enlarges the original pores of microspheres resulting in a larger interaction surface area which is favourable for hemostatic activity. Meanwhile, the exposed HA nanoparticles on the surface of porous microspheres significantly accelerate the blood clotting rate due to their participation in the coagulation cascade. This is due to the adsorption of proteins such as fibrinogen in blood plasma onto HA nanoparticles, followed by platelet adhesion, release reaction, and aggregation. In addition, HA nanoparticles provide high Ca^2+^ concentration surroundings by releasing Ca^2+^, which greatly accelerates the coagulation cascade. The higher HA content not only accelerates the blood-clotting rate, but also attenuates the mechanical strength of microspheres. During the coagulation cascade, the accelerating formed thrombus will be loaded onto the microspheres.

## 5. Tissue Engineering and Regeneration

This section will discuss several areas in which polymer matrix nanocomposite materials have been explored in order to repair and regenerate tissue. In particular the use of cell-containing scaffolds for vessel growth after myocardial infarction, peripheral nerve regeneration, retinal tissue regeneration and neurone regeneration using antioxidant nanoparticles.

Heamostatic activity is important in myocardial tissue engineering. Hydrogels have frequently been studied in this area as bioactive substances that mimic biochemical and biomechanical microenvironment in order to provide a supportive matrix for cell delivery. These materials not only preserve the transplanted cells but also act as physical supports for the thin wall of the heart following MI [103]. One main problem in myocardial tissue engineering is the lack of functional vessels, which ultimately leads to a low survival rate of engineered tissues/injected cells. As a result, angiogenesis potential should be one of the most important characteristics of a scaffold used in this field. Angiogenesis has some major benefits, including delivering oxygen and nutrients to the tissues, removing waste products and the potential to increase engineered construct dimensions. Myocardial tissue engineering after tissue damage occurred from myocardial infarction has explored the use of polymer nanocomposite hydrogel scaffolds to mimic both the biomechanical and biochemical behavior of the tissue whilst supporting cells [104]. Angiogenesis capacity is one of the most important properties of materials for repair in this area. Scaffolds containing bioactive glasses based on SiO_2_, Na_2_O, P_2_O_5_ and CaO have been demonstrated to stimulate angiogenesis and thus the potential for vessel growth into the scaffold [105,106]. Barabadi et al. [104] demonstrated a positive effect on the differentiation of endometrial stem cells into cardiomyocytes when in the presence of a bioactive glass containing hydrogel scaffold.

Another area of interest is the treatment of peripheral nerve damage, which can occur due to various reasons such as an effect of surgical procedures or due to a physical accident. This usually results in atrophy of the muscles surrounding the nerve in question that can lead to partial or complete paralysis [107]. Surgical repair of large nerve defects typically requires the harvesting of a healthy nerve to graft onto the damaged area, known as autografts. This repair technique can often result in damage to the tissue around where the healthy nerve was removed for repair of the damaged site, while another disadvantage is the lack of availability [107]. In order to develop a synthetic biocompatible and biomimetic approach, silk fibroin nanofibers have been used to develop nerve conduits. Two important factors for nerve regeneration are neurite outgrowth and interaction with dorsal root ganglion, silk fibroin that has been functionalised with nerve growth factor has demonstrated a positive effect on both these functions [108]. Furthermore, functionalisation of the silk fibroin with glial-derived nerve factor in addition has been shown to improve the recovery of injured peripheral nerves [109]. Das et al. [107] explored the methods to manufacture nerve conduits. A novel “sheet rolling” method using electrospinning was developed to overcome the dimension limitations of techniques such as electrospinning, moulding and solvent casting which rely heavily on the use of a mould [107]. Das et al. [107] discovered that layer by layer stacking of the nanofibers allows control of the wall thickness of the conduit by altering rotation during electrospinning, and the approach yields low porosity and limited swelling of the conduits. Incorporation of Schwann cells into the nanocomposites served to further enhance their regenerative potential. These developed silk fibroin-gold nanoparticles (GNP-SF) nanocomposite nerve conduit materials were implanted in rats and those with the GNP-SF conduit displayed complete nerve gap growth over an 18 month implantation. When compared to normal silk fibrin conduits, the nerve regeneration was shown to be far superior with the GNP-SF nanocomposite.

Marino et al. [110] have developed gelatin/cerium oxide nanoparticles electrospun into highly aligned nanocomposite fibers. Cerium oxide nanoparticles, also known as nanoceria, are known to be highly effective self-regenerating reactive oxygen species scavengers that possesses the ability to inhibit cell senescence and encourage the development of neurites [110]. Due to the crystalline structural nature of nanoceria, redox activity occurs on the surface of the material with the presence of both Ce^4+^ and Ce^3+^ species [111,112] Ce^3+^ reduces superoxide, thus forming H_2_O_2_ and Ce^4+^, while H_2_O_2_ and Ce^4+^ restores Ce^3+^ and produces O_2_, and this self-regeneration enables nanoceria to effectively control the reactive oxygen species levels within cells [113]. Marino et al. [110] discovered that the gelatin fibres loaded with nanoceria were able to sustain both growth and differentiation of neuronal cells, the antioxidant presence improved neuron phenotypes suggesting the material is ideal for use in nerve guide conduits. There has been a move towards the use of biodegradable nerve conduit materials over non-biodegradable ones, as compression and inflammation of the implantation site that causes inhibition of nerve regeneration is a common side effect when using non-biodegradable conduits [114]. This inflammatory response also occurs with the use of biodegradable conduits, however the presence of an antioxidant such as nanoceria can inhibit such side effects, particularly if they can be released from the scaffold in a controlled release fashion [115]. In particular it is the ability of the nanoceria to self-regenerate that makes it such a suitable antioxidant additive to a nerve conduit material [111,112].

Sephavandi et al. [116] developed a chondroitin sulphate poly(ɛ-caprolactone) CS-PCL copolymer 3-dimensional CS-PCL/PCL scaffold via grafting of ɛ-caprolactone onto chondroitin sulphate hydroxyl groups and combination with PCL for retinal tissue regeneration [117]. This polymer was combined with SrAl_2_O_4_: Eu^2^^+^, Dy^3+^ nanoparticles which were prepared using a standard sol-gel method [118]. Zhao et al. [118] reported that phosphorescence was stimulated by visible light making the particles a suitable inclusion for retina implant materials. The nanoparticles were coated with a PEG solution for biocompatibility, shifting their emitted light from 520 nm to 560 nm, and included within the polymer scaffold generated via electrospinning, in increasing concentrations from 10% to 50% [116]. They reported that a 30% inclusion of nanoparticles within the polymer scaffold displayed the most beneficial biocompatibility and cytotoxicity whilst also displaying adequate mechanical properties [116]. Superior proliferation of neural retinal cells in presence of the 30% SrAl_2_O_4_: Eu^2+^, Dy^3+^/CS-PCL electrospun scaffolds was recorded, demonstrating their suitability for retinal repair and regeneration [116].

## 6. Drug Delivery from Nanocomposites

Drug delivery is one area where nanocomposites can have advantages, and due to the unique nature of their properties some interesting and smart drug delivery systems have been developed. Yolk/shell or ‘rattle-type’ nanomaterials are encouraging nanomaterials for a range of applications such as catalysis, drug delivery, lithium-ion batteries and biosensors due to their adaptability and functionality in both the core and hollow shell [119]. Layered double hydroxides (LDHs) form part of a family of anionic clay materials. These consist of anions layered in between cationic brucite layers. The general composition is M1^−^x^2+^Mx^3+^(OH)_2_ (Ax/nn^−^)·yH_2_O, where M^2+^ and M^3+^ are divalent and trivalent metal cations, respectively, An^−^ denotes an anion, and x the molar ratio of the trivalent cation to the total cation. MgAl-LDHs are suitable materials for drug and or gene delivery as sensitive drugs and genes can often be protected in between interlayers within the structure. The shell of the structure is able to contain catalytic materials which are held within the core of the structure, protecting them from environmental and/or biological systems, whilst allowing diffusion of necessary reactants and subsequent products through the shells [81]. They have low toxicity and various tunable properties such as pH responsiveness, high anion exchange levels and particle size [120].

Other smart materials are shape-memory polymers (SMPs). SMPs are able to regain their original shape after deformation that may occur after an external stimulus. Kashif [121] investigated poly(ε-caprolactone) (PCL)/trisilanolphenyl polyhedral oligomeric silsequioxane (TspPOSS) nanocomposites and their drug release using theophylline as the model drug. The TspPOSS was dispersed throughout the PCL matrix in the form of nanocrystals, which were able to form physical crosslinks with the PCL via hydrogen bonding. The presence of TspPOSS nanocrystals within the PCL/TspPOSS nanocomposites restricted the diffusion of the drug. It was proposed that this may have been due to reactions between silanol groups present in the TspPOSS and carbonyl and/or amine groups of the theophylline. Furthermore, the presence of the TspPOSS within the PCL matrix enhanced the hydrolytic degradation.

Another example is the use of magnetic nanoparticles shown by Long et al. [122] who developed magnetite chitosan/carrageenan nanocomposites. Iron oxide nanoparticles have various current in vivo applications, particularly in contrast agents used in magnetic resonance imaging (MRI), and also as targeted drug delivery, which has great potential, for instance in cancer chemotherapy. Biochemically they are often used in separation processes or immunoassays [123,124]. For the nanoparticles to be effective in their application the surface of the particle must be coated, in particular for the use for sustained drug release where specific particle localization of anchorage is required [125]. Various types of materials have been explored with significance on natural polymers due to their biocompatibility. Long et al. [122] used natural polymeric κ-carrageenan to modify the surface of the magnetite nanoparticles. Another study by Wang et al. [126] used polymeric nanofibers of two cellulose derivatives, dehydroxypropyl methyl cellulose phthalate and cellulose acetate, with magnetic nanoparticles for delivery of indomethacin and aspirin which were used as model drugs. Although we have shown three different methods of how nanocomposites can be used for drug delivery this list is far from exhaustive, as nanocomposites allow for a combination of properties to be included in the same material.

One important area where this unique drug delivery performance is particularly beneficial is in the treatment of cancer. Platinum compounds are an important treatment in cancer chemotherapy. One of the most common anticancer drugs is cisplatin (CDDP), which was one of the first generation platinum-based chemotherapy treatments. Cisplatin is used to treat tumors in various parts of the body such as the head, neck, respiratory, digestive, and renal systems [127,128]. The clinical application of CDDP for cancer chemotherapy is still in limited because of its nonspecific bio-distribution and severe side effects [129]. Raj studied encapsulation of the anticancer drug CDDP into cassava starch acetate/polyethylene glycol/gelatin (CSA/PEG/G) nanocomposites through the interaction between CDDP and CSA/PEG/G nanocomposites. Raj [129] found that the percentage release of CDDP from CSA was slightly greater when compared to that of CSA–PEG and CSA–PEG–G combined nanocomposites. For the CSA–CDDP, CSA–CDDP–PEG and CSA–CDDP–PEG–G coated nanocomposites, the percentage of CDDP released from the nanocomposites was initially much larger and then very slow after some hours. The release rate of the CDDP which was loaded in the CSA, CSA–PEG and CSA–PEG–G loaded nanocomposites was also much lower than the free CDDP. The results indicated that the release of CDDP from CSA, CSA–PEG and CSA–PEG–G nanocomposites is pH dependent, the CDDP released faster in acidic environment than at basic environment as a consequence of binding between drug and the carboxyl group in cassava starch acetate nanocomposites. These results suggest that the CDDP-coated CSA, CSA–PEG and CSA–PEG–G nanocomposites might be used as great potential carriers for controlled drug delivery systems.

Other work on cancer includes the research of Schweta et al. [130] who investigated a lignin-tetraethoxysilane-based nanocomposite to evaluate its antimicrobial activity and cytotoxicity in human cancer cells. They were able to demonstrate efficient antibacterial activity against various strains, which would be modified by varying concentrations of lignin and silica. The proposed mechanism of this antibacterial activity was deemed to be attributable to the nanocomposites’ ability to enter the bacterial cell—the lignin-TEOS nanocomposites were 1–20 nm in size in comparison to a bacterial cell of 100–1000 nm in size. Upon entering the cell the nanocomposite may interact with hydroxyl and or carboxyl groups and potentially any other surface functional groups in the cell and deactivate their function via the release of silicate ions. It may combine with respiratory enzymes to induce asphyxiation and inhibition of cell replication, which subsequently leads to cell death [131].

The use of nanomaterials for simple drug delivery has already been highlighted in this section, as well as some more smart delivery systems. A multifunctional smart nanomaterial, may be loaded with both therapeutic drugs and diagnostic agents into one single material, this type of multifunctional material is defined as a theranostic material. Ho et al. [132] proposed an innovative polymeric nanoparticle bilayer material with a core-shell structure. The bilayer type structure allows the multifunctional nature of the material, which was termed “unibody core-shell (UCS)”. This UCS is Gd^3+^ at the core and doxorubicin at the shell to simultaneously allow MRI contrast and cancer drug release.

## 7. Conclusions

This review paper has demonstrated that nanocomposites have drawn considerable research interest in bio-applications. This is due to the promise of their wide range of properties and benefits. One of the earliest bioactive nanocomposite materials developed was bioglass. This still remains one of the most common used nanocomposite materials, particularly in bone tissue engineering uses. However, with advances in immunohistochemistry analyses and the identification of relevant markers of osteogenesis and growth factors important for ECM regeneration, bioactive bioglass nanocomposites are increasingly being demonstrated as beneficial and used in other body sites. Drug delivery has seen the usage of many ‘smart’ and unique systems, for example, core-shell-based materials have allowed significant advances in drug delivery allowing the targeted delivery of effective compounds which previously could not access the site of interest alone. These new “smart” materials which can provide both a diagnostic and therapeutic response are at the forefront of developments in nanocomposite materials for drug delivery applications. The capacity to include highly antioxidant nanoparticles within a scaffold material for tissue regeneration is a significant advance in the potential to encourage tissue which has sustained significant injury to heal, scavenging oxidative species produced in a response to trauma, providing cells with an advantageous environment for healing. Advances in manufacturing capabilities such as electrospinning, sheet deposition methods, etc. have allowed the development of more novel nanocomposite materials. This is where much of the future trends in this area will arise, with greater capabilities to control physical and chemical properties of the nanocomposites. Greater control of structure will enable the interactions with body sites to be assessed in more detail and thus provide further advances in this area. However, although nanocomposites and bioactive materials have great potential, there is one area of concern in the synthesis and subsequent therapeutic use in other biological environments; there is the lack of knowledge relating to the toxicity of nanoparticles within the human body. Several parameters are reported to be key in the subsequent toxicity of a material, size, crystalline structure, chemical composition, surface area and oxidation state [133]. All of these concerns need to be addressed before we see further day to day use of nanocomposites in soft tissue repair and regeneration.
